# An MRI-Based Method for the Morphologic Assessment of the Anterior Tibial Tuberosity

**DOI:** 10.3390/jcm13216601

**Published:** 2024-11-03

**Authors:** Emi Marinela Preda, Nicolae Constantin, Serban Dragosloveanu, Romica Cergan, Cristian Scheau

**Affiliations:** 1 Department of Radiology, The “Carol Davila” University of Medicine and Pharmacy, 050474 Bucharest, Romania; emi.preda@umfcd.ro; 2 Department of Radiology and Medical Imaging, “Foisor” Clinical Hospital of Orthopaedics, Traumatology and Osteoarticular TB, 021382 Bucharest, Romaniacristian.scheau@umfcd.ro (C.S.); 3 Department of Radiology and Medical Imaging, Bucharest Emergency University Hospital, 050098 Bucharest, Romania; 4 Department of Orthopaedics and Traumatology, The “Carol Davila” University of Medicine and Pharmacy, 050474 Bucharest, Romania; 5 Department of Orthopaedics, “Foisor” Clinical Hospital of Orthopaedics, Traumatology and Osteoarticular TB, 021382 Bucharest, Romania; 6 Department of Anatomy, The “Carol Davila” University of Medicine and Pharmacy, 050474 Bucharest, Romania; 7 Department of Physiology, The “Carol Davila” University of Medicine and Pharmacy, 050474 Bucharest, Romania

**Keywords:** MRI, X-ray, anterior tibial tuberosity index, Osgood-Schlatter, morphometry, anatomy, protocol, method comparison

## Abstract

**Background**: A prominent anterior tibial tuberosity (or tibial tubercle) can be seen in ongoing Osgood–Schlatter disease (OSD) in teenagers or as a sequela of OSD in adults. Current radiological methods do not provide a true anatomical assessment of the tibial tuberosity; therefore, we proposed and developed a Magnetic Resonance Imaging (MRI)-based method for measuring the anterior tibial tuberosity index, aiming to deal with the current lack of effective techniques for accurately assessing these particular morphologic features. **Methods**: A retrospective study included 47 knees with tibial tuberosity measurements on both true sagittal MPR images of 3D proton density (PD)-weighted MRI sequences and lateral knee radiographs. The same landmarks were followed and the anterior tibial tuberosity index (ATTI) was measured. **Results**: The comparison of the results obtained by the two methods demonstrates that our method is reliable and reproducible with substantial inter- and intra-observer agreement. The intraclass correlation coefficient was 0.9250 (95% CI: 0.8654 to 0.9582), indicating excellent reliability between the two methods. A strong positive correlation was also identified, with a correlation coefficient of r = 0.8746 (95% CI: 0.7845 to 0.9286, *p* < 0.0001) between the two methods. No significant deviation from linearity was observed by analyzing the linear model validity using the cusum test (*p* = 0.62). **Conclusions**: Based on these results, we encourage the use of 3D PD-weighted MRI sequences for the measurement of the anterior tibial tuberosity on MRI in order to avoid unnecessary exposure to ionizing radiation and potentially obtain a more accurate measurement. Future larger studies should also explore the benefit of utilizing 3D sequences over 2D lateral projections to minimize measuring bias.

## 1. Introduction

The anterior tibial tuberosity may appear prominent due to ongoing Osgood–Schlatter inflammation in adolescents or as a sequela of Osgood–Schlatter disease (OSD) in adults [1,2]. Symptoms can persist into adulthood; however, the sequelae of the disease are rarely imaged, usually only on radiographic examinations, and feature a prominent anterior tibial tuberosity and irregularities at this site or the presence of some bone fragments in the tendon, at the tibial insertion [1,3].

Several types of surgery are therefore described in the literature in patients with OSD or OSD sequelae, either endoscopic or surgical, in an attempt to reduce persistent symptoms. Some authors suggest the reduction of patellar tendon irritation and symptoms together with the dimensional reduction of the tibial tuberosity [4,5]. Although the literature studies report the reduction of the tibial tuberosity postoperatively, we have not found any study that discloses the modality of pre- and postoperative measurements of the tibial tuberosity itself [6,7,8,9]. Most of these surgical studies use indirect radiological measurements such as the Caton–Deschamps index for patellar height as pre- and postoperative indices [7]. Also, to our knowledge, there is no minimal classification of the dimensions and morphology of the tibial tuberosity to help monitor its surgical modeling.

Even though both computed tomography (CT) and Magnetic Resonance Imaging (MRI) provide three-dimensional data and could be useful in identifying the location of ossicles [10], MRI is the exam of choice for pre- and postoperative imaging of the knee, where the condition of the tendon and the tibial tuberosity can be evaluated in great detail [2,7,11]. However, the only apparently detailed method of measuring tibial tuberosity found in the literature is performed on the lateral view knee radiograph by Visuri et al. [12]. While radiographic methods have been the cornerstone of musculoskeletal imaging due to their cost-effectiveness and general accessibility, they show limitations in the context of assessing the tibial morphology, in particular the anterior tibial tuberosity, due to their two-dimensional nature [13]. Also, the method is highly dependent on the skill and experience of the technician and may be significantly influenced by an altered or improper patient positioning [14].

In contrast, MRI offers significant advantages in overcoming these limitations and is still underutilized in clinical practice for this specific role. As a non-invasive and non-irradiating method, MRI provides detailed three-dimensional (3D) images and permits 3D reformatting and reconstructions to select adequate positioning planes for measurement. Unlike radiographs, MRI is not dependent on perfect patient positioning, as the image planes can be reformatted after the scan to align with the ideal anatomic orientation, which is crucial for the proper identification and reporting of ossicles or other irregularities around the tibial tuberosity. Due to these considerations, MRI would therefore be superior and would lead to a decrease in unnecessary irradiation in these patients. However, due to its higher cost and restricted accessibility in certain cases, MRI is generally reserved for complex cases [15]. This gap in practice, where radiographs still dominate as the elective diagnostic method despite their limitations, underlines the need to develop a standardized MRI-based method such as the one presented here.

In our study, we aimed to propose, describe, and validate an MRI method for measuring the tibial tuberosity and to compare the results with radiographic measurements in the same patients. We hypothesized that no significant differences between the methods would be identified, therefore the methods would be comparable. 

## 2. Materials and Methods

After obtaining institutional review board approval, we retrospectively analyzed the patients at our hospital who underwent both an X-ray and MRI of the knee. The patients were recruited from our hospital electronic database, with the following criteria of inclusion: patients over 18 years old without fractures, tumors, or surgical interventions on the knee region. The database was screened for the period August 2023–June 2024. A total of forty-one patients were identified, of which six had bilateral examinations, resulting in a total of forty-seven pairs of X-ray and MRI investigations. We included patients who underwent MRI investigations secondary to radiographic examinations and we did not submit any patient to unnecessary X-ray exposure. The MRI investigations were performed for radiographic-negative persistent knee pain with or without previous trauma and revealed meniscal pathology and/or ligament tears in most cases (*n* = 39). In the remaining cases (*n* = 8), the knees were within the normal limits. All patients signed an informed consent form when undergoing the imaging procedures.

The knee radiographs were performed on a DigitalDiagnost R3.1 machine (Philips Medical Systems Nederland B.V, Amsterdam, The Netherlands). The lateral view was performed with a focus-film distance of 115 cm, in lateral decubitus, with a slight flexion of 20 to 30°. The examined knee was laid on the table and the contralateral leg was placed anteriorly with flexion of the hip. The tube was tilted 5 to 7° and the beam was centered at the knee joint and on the midline, and collimated to include the distal femur, proximal tibia and fibula, and skin margins anteriorly and posteriorly. Exposure parameters were 66 kV and 5 mAs.

**Figure 1:**
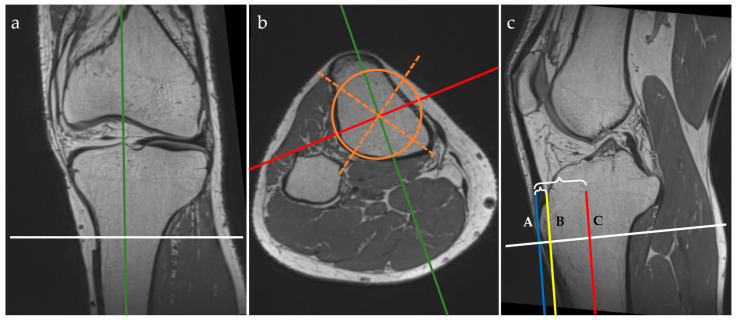
Three-plane view of multiplanar reformatting in the coronal (**a**), axial (**b**), and sagittal (**c**) planes of the 3D PD isovolumetric acquisition and graphical overlay representation of the MRI protocol for the anterior tibial tuberosity index (ATTI) assessment. The lines represent the following: tibial midline sagittal plane (green), coronal plane through the middle of the tibia (red, C), axial plane (white), geometric tibial axes (orange dotted lines) and area (orange circle). Tangent lines through the tuberosity of the anterior tibia (blue line, A), and through the epiphyseal line of the anterior tibial cortex (yellow line, B). Distances between A and B, and A and C, respectively, are marked with accolades.

All MRI examinations were performed with a Magnetom Vida 3T machine (Siemens, Munich, Germany) using the dedicated knee MRI protocol of the Department of Radiology and Medical Imaging within the “Foisor” Clinical Hospital of Orthopaedics, Traumatology and Osteoarticular TB. The knee examinations were performed with the dedicated knee coil (TxRx Knee 18) with the patients positioned supine, feet first. The studied knee was immobilized with positioning sponges and sand bags while the contralateral knee was placed on top of a dedicated support. All patients benefited from a 3D PD-weighted isovolumetric sequence acquired in the sagittal plane (resolution = 0.5 × 0.5 × 0.5 mm, TR = 1200 ms, TE = 33 ms, flip angle = 120 degrees, bandwidth = 422 Hz/Px, and scanning time = 5 min and 31 s), which is part of the routine knee protocol in our department. The postprocessing was performed using the multiplanar reformatting (MPR) view mode of the console software, where the actual measurements were taken, as in the example in Figure 1.

On the MPR view, landmarks were drawn as follows:

- on the coronal plane: we aligned the vertical axis with the middle of the tibial shaft and the horizontal axis parallel with the epiphyseal line and passing through the maximum point of the tibial tuberosity;
- on the axial plane: we aligned the axes with the middle of the tibial shaft (both the sagittal and coronal axes) by drawing a circle that fitted the cross-section of the tibial shaft and attempted to overlie the posterior cortical line while covering the maximum surface, then drawing two intersecting lines within the circle to find the geometrical center; the sagittal axis was then rotated to pass through the maximum point of the tibial tuberosity;
- on the sagittal plane: we aligned the horizontal axis with the epiphyseal line.

When using the MPR view, the three planes (i.e. coronal, axial, and sagittal) were orthogonal. The next steps were to draw vertical lines, as follows:

- we identified the most prominent point of the tuberosity of the anterior tibia and drew a vertical line tangent to this point—the blue line (A);
- we identified the anterior tibial cortex at the level of the epiphyseal line and drew another vertical line tangent to this point—yellow line (B);
- these two vertical lines must be parallel to the vertical axis that passes through the middle of the tibial shaft—the red line (C).

We measured the distances between these three lines to obtain the anterior tibial tuberosity index (ATTI) and we calculated the ratio between them, dividing the distance between A and B by the distance between A and C (i.e. (AB)/(AC)). This represents a transposition of the method for measuring the anterior tibial tuberosity index on the lateral radiographs of the knee, as described by Visuri et al. [3,12].

**Figure 2:**
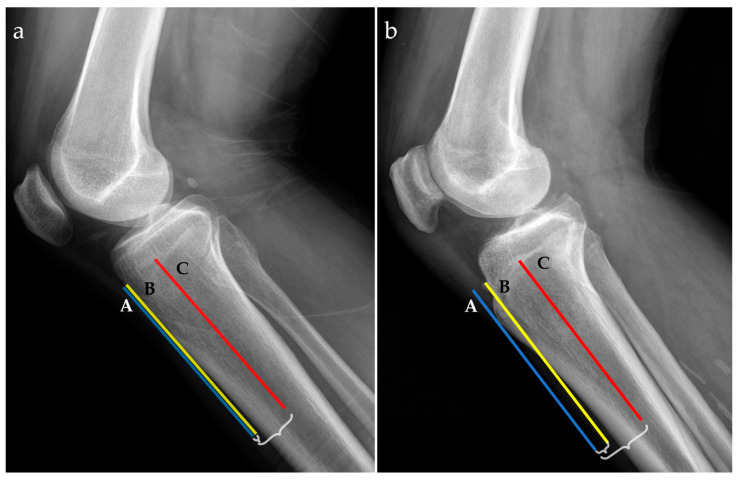
Radiographic lateral views for two patients with low (**a**) and high (**b**) anterior tibial tuberosity index (ATTI). The following landmarks are represented: a tangent line at the most prominent point of the anterior tibial tuberosity (blue line, A), the anterior tibial cortex (yellow line, B), and the middle tibial diaphysis (red line, C). Distances between A and B, and A and C, respectively, are marked with accolades.

For radiographic examinations (Rx), the ATTI was obtained by the ratio of two distances; the first one measured between the anterior tibial cortex and a tangent line through the most prominent anterior point of the tibial tuberosity and the second one between the middle of the tibial diaphysis and a tangent line through the most prominent anterior point of the tibial tuberosity, as exemplified in Figure 2.

For the statistical analysis, we assessed patient age and gender, as well as numeric measurements of distances on both the MRI and radiographic images. We tested the data for normal distribution by using the Shapiro–Wilk method. For continuous variables, we used the average ± standard deviation to express the values. Student’s t-test was used to compare the mean values between the two types of measurements. To compare the two methods, we used the Bland–Altman plot and the Passing and Bablock regression. We also quantified the reliability of the MRI-based method compared to the radiographic measurement by calculating the intraclass correlation coefficient for both the single and average measures. Pearson’s correlation coefficient was also computed to examine the linear relationship between the two methods. Two observers measured independently, with repeated measurements being performed two weeks apart, and the reliability was assessed using Cohen’s Kappa coefficient. The analysis was performed in MedCalc® Version 14.8.1 (MedCalc Software bvba, Ostend, Belgium). For this study, we considered that results were statistically significant for *p*-values less or equal to 0.05.

## 3. Results

The average age of the patients was 40.27 ± 15.33 years, with a normal distribution. This study included 24 men and 17 women with a male-to-female ratio of 1.47:1. In terms of laterality, 30 (63.8%) of the total examinations were on right knees, while 17 (36.2%) were on left knees. Additionally, six patients provided bilateral data.

A general analysis of the reported MRI and radiographic data showed a normal data distribution with mean values of 0.1700 ± 0.04408 for MRI and 0.1751 ± 0.05283 for Rx. No significant difference between the values was noted (*p* = 0.6121), suggesting a comparable level of precision between the MRI and radiography for this purpose.

Furthermore, to assess the agreement between the MRI and radiographic measurements of the anterior tibial tuberosity we calculated the intraclass correlation coefficient (ICC) using a two-way model for consistency. We obtained an ICC (average measures) of 0.9250 (95% CI: 0.8654 to 0.9582), indicating excellent reliability between the two methods. The single-measures ICC was also high, at 0.8605 (95% CI: 0.7628 to 0.9198). 

A Pearson’s correlation coefficient was calculated to assess the linear relationship between the anterior tibial tuberosity measurements obtained through the MRI and radiographs. The results demonstrated a strong positive correlation between the two methods, with a correlation coefficient of r = 0.8746 (95% CI: 0.7845 to 0.9286), indicating a significant relationship between the two variables (*p* < 0.0001).

**Figure 3:**
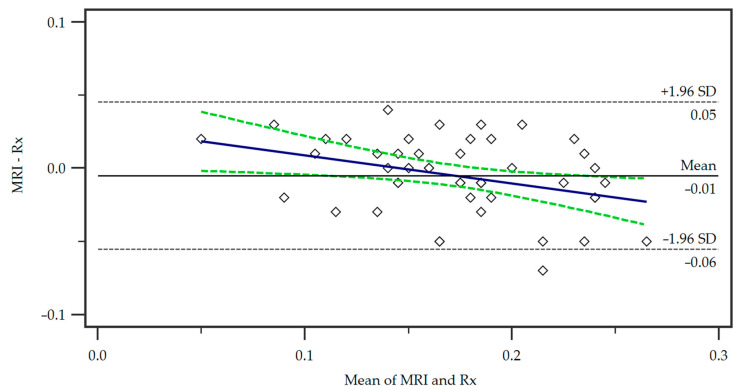
Bland–Altman plot of the differences in the measurements of the two methods, Magnetic Resonance Imaging (MRI) and radiography (Rx). A regression line of differences is presented (blue line) as well as the associated 95% Confidence Interval (dotted green lines). SD = standard deviation.

For the comparison of our proposed MRI protocol with the standard radiographic measurement, we employed a Bland–Altman plot (Figure 3).

The mean of the measurements from the two methods is represented on the x-axis, while the y-axis shows the difference between the measurements (MRI minus radiograph). The central horizontal line shows the mean difference or bias between the methods, which in this case was small, indicating no systematic over- or underestimation of tibial tuberosity size by either method. The distribution of the points within the 95% limits of agreement (+/−1.96 times the standard deviation) shows that the majority of the data points were located within an acceptable range of agreement. No significant trend in differences was observed demonstrating a consistent agreement of the methods.

**Figure 4:**
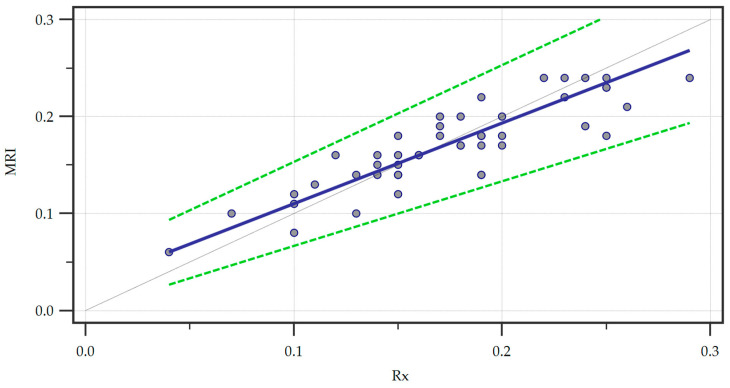
Passing and Bablock regression of the two methods, Magnetic Resonance Imaging (MRI) and radiography (Rx). A regression line (blue line) is featured alongside confidence interval curves (dotted green lines).

Furthermore, we ran a Passing and Bablock regression to explore the linear relationship between the measurements obtained through the two methods (Figure 4). 

Within the sample size, no significant deviation from linearity was observed by analyzing the linear model validity using the cusum test (*p* = 0.62). We also recorded the systematic (intercept A = 0.02667, 95% CI = 0.0000–0.05333), proportional (slope B = 0.8333, 95% CI = 0.6667–1.0000), and random (residual standard deviation = 0.01725, ± 1.96 interval = −0.03382–0.03382) differences. The intercept A yielded by the regression suggested a small systematic difference between the two methods and since the confidence interval for the intercept included zero there was no significant systematic bias. Furthermore, the confidence interval of the slope B included one indicating no statistically significant proportional bias. Finally, the small residual standard deviation confirmed that the measurements of the two methods were very similar with minimal random variation.

Regarding the reliability assessment of the measurements, inter-observer agreement was 0.734 for MRI measurements compared to 0.758 for Rx, and intra-observer agreement was slightly higher, with values of 0.795 for MRI and 0.845 for Rx.

## 4. Discussion

Our study aimed to develop an MRI-based method for measuring the ATTI and to compare the results obtained with those reported by Visuri et al. using lateral radiographs [12]. We obtained substantial agreement between repeated measurements for calculating the ATTI using MRI. Analyzing the results, the high ICC values, strong Pearson’s correlation, consistent agreement shown in the Bland–Altman analysis, and lack of bias indicated by the Passing and Bablock regression, demonstrated that the MRI-based measurement of the ATTI was highly reliable and comparable to standard radiographic techniques. These findings support the use of MRI as a suitable alternative to radiography, with the added benefits of three-dimensional visualization and the elimination of ionizing radiation exposure.

In our study, the anatomical landmarks were described utilizing a 3D isovolumetric PD-weighted sequence which was part of our department’s routine protocol. For optimal visualization of the cortical bone and more accurate measurements, we recommend scanning in the sagittal plane of a PD-weighted 3D sequence and suggest extending the acquisition FOV at least 2 cm below the lowest point of the patellar tendon insertion. All measurements should be performed on the MPR views. The three-dimensional nature of the images is essential for obtaining high-quality reformatted images, and isovolumetric (isotropic) sequences are preferred in this regard; however, since no particular lesion or structure other than the skeletal elements are analyzed, other types of 3D sequences may also be utilized (T1 or T2-weighted) as long as sufficient detail is provided to accurately perform the measurements [16,17]. This might include sequences that are part of the routine knee protocol in various centers. However, certain advantages of 3D PD sequences over T1 and T2-weighted imaging (WI), were noted as follows: 3D T1 spin echo sequences may have long acquisition times while faster techniques, such as Dixon, may have insufficient resolution for proper reformatting; on a different note, 3D T2 WI may yield chemical shift artifacts and have insufficient detail on bone contours, besides the extended scanning duration [18,19]. In this context, we believe that the 3D PD-weighted sequence is the best choice in this particular type of measurement.

Although the pathophysiology of OSD is unclear and there are still discussions and uncertainties about how hypertrophic tibial tubercles should be approached in patients with ongoing disease or its ensuing sequelae, there are some authors who consider that chronic distal patellar tendinosis is caused by the prominent tibial tubercle itself, recommending its osteotomy/remodeling, thus leading to the disappearance of the patellar tendon irritation [4,5,6,20].

While increasingly great efforts to reduce medical radiation exposure are visible worldwide through initiatives such as ALARA (“as low as reasonably achievable”), X-rays and CT scans continue to play essential roles in current orthopedic practice [21,22]. X-rays remain critical for the initial assessment of fractures, staging of osteoarthritis, and the evaluation of joint alignment, as well as for preoperative digital planning [23,24,25,26]. Meanwhile, CT imaging is also crucial for assessing complex fractures, 3D planning, and acquiring volumetric data for 3D printing applications or robotic surgery tracking or navigation [27,28,29,30,31,32]. While MRI has made considerable advancements in proficiency in these advanced orthopedic applications, X-rays and CT scans continue to play important roles even though efforts to reduce radiation exposure remain a priority [33].

In perhaps the most extensive review of the methods used to measure the patellar height, considered to be the basic criterion for the management of several pathologies of the anterior compartment of the knee, including OSD, from 1938 to 2019, Igomenou et al. conclude that none of the proposed methods are still accepted as the gold standard. Also, the authors conclude that MRI methods are becoming more and more promising but will need to be proven in large studies [34]. Therefore, the measurement of other parameters, including the patellar tendon length with MRI, has already proven its effectiveness in determining the position of the patella [35,36].

While lateral projection X-rays have been the commonly used imaging modality to measure the anterior tibial tuberosity [12], MRI is an increasingly available and non-irradiating modality, as opposed to X-ray and CT. MRI is used for surgical planning and brings several diagnostic benefits, such as 3D imaging capabilities, improved tissue contrast, detailed assessment of regional anatomy, and the capability of detecting numerous other knee pathologies [7,37]. 

Further evidence of the increasingly important role of MRI in orthopedic imaging is emphasized by the advancements in sequences and modalities that allow for detailed and precise visualization of bone structure and morphology. While traditional MRI has demonstrated an invaluable role in soft tissue assessment, newer techniques such as ultrashort echo time (UTE) and zero echo time (ZTE) imaging have expanded MRI capabilities to include accurate imaging of the cortical bone [38,39,40]. Consequentially, these new sequences can facilitate the assessment of bone integrity and microarchitecture. Furthermore, high-resolution 3D images allow for the planning of interventions and evaluation of postoperative bone healing, minimizing the role of X-ray-based imaging and making it a good instrument in the field of personalized orthopedics [40,41]. While MRI seems like a more costly method compared to X-rays, it might be more cost-effective when considering the potential for reducing repeated imaging due to its superior accuracy as well as limiting possible radiation-exposure complications [42].

Our study has several limitations including the small number of studied cases, where both the MRI and radiographs were available in the same patient. Further studies with a larger number of enrolled patients are necessary to reinforce the validity of this method. Also, a difference in accuracy between the compared methods may exist, due to the variability introduced by discrepancies in the patient positioning during imaging. On the lateral X-ray view, the knee is flexed at 20 to 30 degrees, while on MRI the flexion is obtained at 15 degrees due to the limitation of the knee coil. Rotational errors in positioning the knee for lateral radiographs could also represent a source of error in measuring the anterior tibial tuberosity index. Nevertheless, the 3D capabilities of MRI provide the best visualization of the local anatomy from various angles, thereby enhancing the measurement accuracy.

In spite of these limitations, we believe that our work makes an important contribution to the diagnostic algorithm and the monitoring of patients treated surgically by remodeling the tibial tuberosity, especially in the case of patients with OSD, but with the potential for use in other situations, such as in various patellofemoral disorders. Further research and contributions are needed to outline a state-of-the-art preoperative assessment, considering not only the type of treatment to be applied but also the diagnostic and monitoring techniques used. While the clinical status and patient outcome were outside the scope of our paper, it is important that future studies assess the relevance of the described method of ATTI measurement in the clinical evolution and surgical results and evaluate whether it may serve as a predictor in this regard. Future research could also reveal improvements and refinements of the method that could further increase its reproducibility and consistency of measurements. Finally, the integration of artificial intelligence in the assessment of the bony morphologic parameters could enhance measurement precision and automate the assessment of knee anatomy with the potential of decreasing the variability between measurements and improving the consistency of the method.

## 5. Conclusions

In the spirit of reducing diagnostic radiation and using a short and fast MRI protocol, the results obtained in our study demonstrate that our proposed method for measuring the anterior tibial tuberosity on MRI is reliable and reproducible and may be used as an alternative method to conventional X-ray assessment.

## Data Availability

The datasets used and/or analyzed during the current study are available from the corresponding author upon reasonable request.
